# Built Environment Audits Across Space and Time for Estimating Time-Varying Exposures: Cohort Study

**DOI:** 10.2196/86279

**Published:** 2026-08-19

**Authors:** Fangyi Wang, Xinyi Xu, Elizabeth Ghias, Stephen J Mooney, Andrew G Rundle, Wilmot A Sowa, Kevin Henry, Antoinette Stroup, Gerald Harris, Jie Li, Peter P Stanich, Heather Hampel, Jesse J Plascak

**Affiliations:** 1 Department of Statistics, The Ohio State University, Columbus, OH, United States; 2 The Ohio State University Comprehensive Cancer Center–Arthur G. James Cancer Hospital and Richard J. Solove Research Institute, 3650 Olentangy River Rd, Columbus, OH, 43214, United States, 1 6142938024; 3 University of Washington, School of Public Health, Seattle, WA, United States; 4 Columbia University, Mailman School of Public Health, New York, NY, United States; 5 Department of Geography, Temple University, Philadelphia, PA, United States; 6 Cancer Prevention and Control Program, Rutgers Cancer Institute, New Brunswick, NJ, United States; 7 Department of Medicine, The Ohio State University, Columbus, OH, United States; 8 City of Hope, Department of Medical Oncology and Therapeutics Research, Duarte, CA, United States

**Keywords:** neighborhood disinvestment, spatiotemporal universal Kriging, colorectal cancer, survival analysis, Google Street View imagery

## Abstract

**Background:**

Neighborhood disinvestment, characterized by built environment disrepair and deterioration, has been linked to health behaviors and outcomes, including cancer survival. However, disinvestment temporal dynamics, including time-lagged exposure estimates among colorectal cancer (CRC) cases, remain underexplored.

**Objective:**

This study aimed to describe and validate a spatiotemporal neighborhood audit protocol using Google Street View imagery, develop and compare predictive spatiotemporal models of neighborhood disinvestment, and examine time-lagged associations between disinvestment and CRC survival.

**Methods:**

We conducted 8256 virtual audits of Franklin County, Ohio, streetscapes sampled across locations and dates from 2009 to 2022 using 7 disinvestment indicators: garbage, graffiti, abandoned buildings, building conditions, yard conditions, road verge conditions, and large dumpsters. Of these, 5751 eligible location date audits were included. A neighborhood disinvestment score (NDS) was derived using item response theory. We fit spatiotemporal regression Kriging models with a simplified sum-metric covariance structure and compared two candidate models using out-of-sample root mean square prediction error (RMSPE): (1) a model incorporating major highways, waterways, and railways to define neighborhood boundaries, and (2) a traditional Kriging model allowing NDS to vary continuously across space and time. The best-fitting model was used to estimate NDS at the geocoded address and diagnosis date of 2727 CRC cases diagnosed from 2012 to 2019 and recorded within the Ohio Cancer Incidence Surveillance System. Covariates included age, sex, minoritized race-ethnicity, marital status, health insurance, and cancer stage at diagnosis (localized, regional, and distant). We built accelerated failure time models to estimate time ratios and 95% CIs for NDS averaged over 0-, 3-, 6-, 12-, 18-, and 24-month time lags. Models were adjusted for covariates. We tested stage by NDS interactions. Those alive through December 31, 2020, were right censored.

**Results:**

NDS exhibited substantial spatial but modest temporal variation, with spatial correlation measurable within 2.8 kilometers and temporal correlation up to 270 days. Traditional spatiotemporal Kriging outperformed the boundary-based model (RMSPE_traditional_=0.651 vs RMSPE_boundary_=0.662). NDS varied by season, with lower disinvestment scores in spring and summer compared with fall. The accelerated failure time CRC survival model indicates an interaction between NDS and stage at diagnosis for all NDS time lags tested; higher prediagnosis NDS was significantly associated with shorter survival only among those with regional and not among those with localized or distant stage at diagnosis. For example, with a 6-month prediagnosis averaged time lag, each standard deviation increase in NDS was associated with a time ratio of 0.628 (95% CI 0.436-0.904). Associations were largely identical across time lags.

**Conclusions:**

The spatiotemporal neighborhood audit approach is feasible and efficient. NDS varies appreciably across season, was accurately estimated using regression Kriging, and time-lagged exposures from 0 to 24 months produced negligible change in the NDS-CRC survival association.

## Introduction

Less walkable and more disinvested built environments have been associated with reduced physical activity; increased sugar-sweetened beverage consumption; higher BMI; more use of tobacco, alcohol, and other psychoactive substances; and various negative health outcomes including lower quality of life, poorer mental health, and shorter cancer survival [1-7]. Numerous studies have used georeferenced and publicly available Google Street View (GSV) imagery (since 2007) to assess observed built environment characteristics of participants’ residential address, which for large samples and in-person assessments would otherwise be infeasible [8]. There are two competing explanations for correlations between disinvestment and health behaviors: (1) a causal mechanism via the psychosocial stress pathway, and (2) unaccounted biasing factors that are causally related to both disinvestment and the given health behavior or outcome (individual socioeconomic factors, organizational resource availability, health care access and receipt, social cohesion, selection into neighborhoods, etc) [2,9-11]. Built environment disinvestment measurement could influence results of studies with health outcomes, yet such considerations are mostly unexplored.

The spatial characteristics of built environment sampling schemes and audit item observations have received far more attention than the temporal characteristics. For example, principles of spatial sampling have been explored in detail in relation to studies of built environment factors and outdoor physical activity [12]. Because sampling efficiency affects precision of exposure pattern estimates [13], many studies report spatial autocorrelation statistics—magnitude and distance at which observations correlate—of measured built environment factors. For example, an early study of “landscape mimicry” found that as much as 50.9% of various residential landscape characteristics (eg, presence or absence of annuals, perennials, trees, shrubs, flower boxes; flower colors, yard condition) were shared across a neighborhood, and that landscaping was statistically significantly more similar up to 7 parcels of separation [14]. Attributes of built environment disinvestment, including excessive garbage, graffiti, abandoned buildings, and building conditions, have also been found to spatially autocorrelate between 1 and 10 kilometers across a study region [15]. However, fewer empirical investigations have explored temporal variation or autocorrelation of built environment characteristics.

Testing and reporting temporal variation in neighborhood disinvestment could support the design of longitudinal public health studies, yet the feasibility of collecting such exposure data from GSV is not well documented. Over 15 years of GSV historical imagery are available, and the long latency suspected for chronic stress-related exposures of health outcomes points to the need for exposure measurements across time [4]. Furthermore, numerous neighborhood investment initiatives and policies that are spatially targeted to change built environment disinvestment characteristics across various timescales—economic tax incentives for “revitalization,” abandoned building demolition policies, property code violation and enforcement, and neighborhood litter pickup and related initiatives—suggest that built environment disinvestment indicators may exhibit temporal, in addition to spatial, variation and autocorrelation [16,17]. Finally, evidence of residential mobility among participants of epidemiologic studies suggests that participants' residential neighborhoods are not static [18-20]. For example, 1 study among New York state mesothelioma cases diagnosed from 2011 to 2015 found that cases moved an average of 3 times up to 15 years before diagnosis and that time-weighted air toxic exposures varied by several covariates, including race-ethnicity [21]. Characterizing temporal variation in neighborhood disinvestment across fixed locations is understudied and important for designing future longitudinal public health studies and, specifically, whether disinvestment measures repeated across time are needed or not.

To support these efforts and address the aforementioned measurement gaps, we extended previously validated audit methods and spatial sampling schemes to assess built environment characteristics retrospectively across both locations and dates using GSV imagery, with a primary focus on neighborhood disinvestment. The study was guided by three research questions: (1) Are historical GSV imagery available for a retrospective spatiotemporal audit, and what limitations arise from the availability and distribution of imagery across locations and dates? (2) How does neighborhood disinvestment vary across space, time, and season, and does explicitly modeling continuous spatiotemporal dependence improve out-of-sample prediction relative to spatial-only and discrete-based (neighborhood boundaries) alternatives? (3) Is greater neighborhood disinvestment associated with colorectal cancer (CRC) survival, and do these associations differ by stage at diagnosis or across exposure-averaging windows extending from the date of diagnosis (0 month) to 24 months before diagnosis?

## Methods

### Study Region and Audit Sample

The study region is Franklin County, Ohio. Franklin contains Columbus—the second most populous city in the Midwestern United States—and has a large land area (1379 km^2^) and a mix of urban, suburban, and open space or rural-type land use (ie, some census tracts have Rural Urban Commuting Area codes other than “Metropolitan Area Core”) [22].

We randomly generated 3535 locations for auditors to assess GSV imagery. The minimum, average, and standard deviation distance in meters between each candidate location and its nearest neighbor was about 51, 259, and 168, respectively. At each location, auditors assessed imagery from up to 5 dates: earliest (limited to 2009 or later), most recent, middate between earliest and recent, 25th percentile between earliest and most recent, and 75th percentile between earliest and most recent. Date quantiles were selected with the goal of achieving a uniformly distributed sample of audits across time.

### Audit Protocol and Measures

Items assessed were selected from previously validated neighborhood audit tools [23]. The audit protocol assessed 3 constructs: disinvestment from 7 items (garbage, graffiti, abandoned buildings, building condition, yard condition, dumpsters, and road verge condition), engagement from 4 items (team sport equipment, outdoor seating, yard decorations, potted plants), and sidewalk walkability from 2 items (walkway type and walkway condition).

Six paid undergraduate research assistants were trained in a 1-hour orientation session, during which they were introduced to the study’s goals, objectives, and key construct definitions, followed by a 4-hour session in which they were introduced to the CANVAS (Computer Assisted Neighborhood Visual Assessment System) audit platform and audit protocol [23]. Auditors were trained on 75 non-Franklin County locations in Ohio. A training manual that also served as a reference guide during auditing was provided. Auditing of the candidate study location dates began only after adequate agreement was achieved across the 75 test locations. Table S1 in Multimedia Appendix 1 summarizes each audit item by name, full wording, response options, and response frequency.

### Data Collection

**Figure 1.**
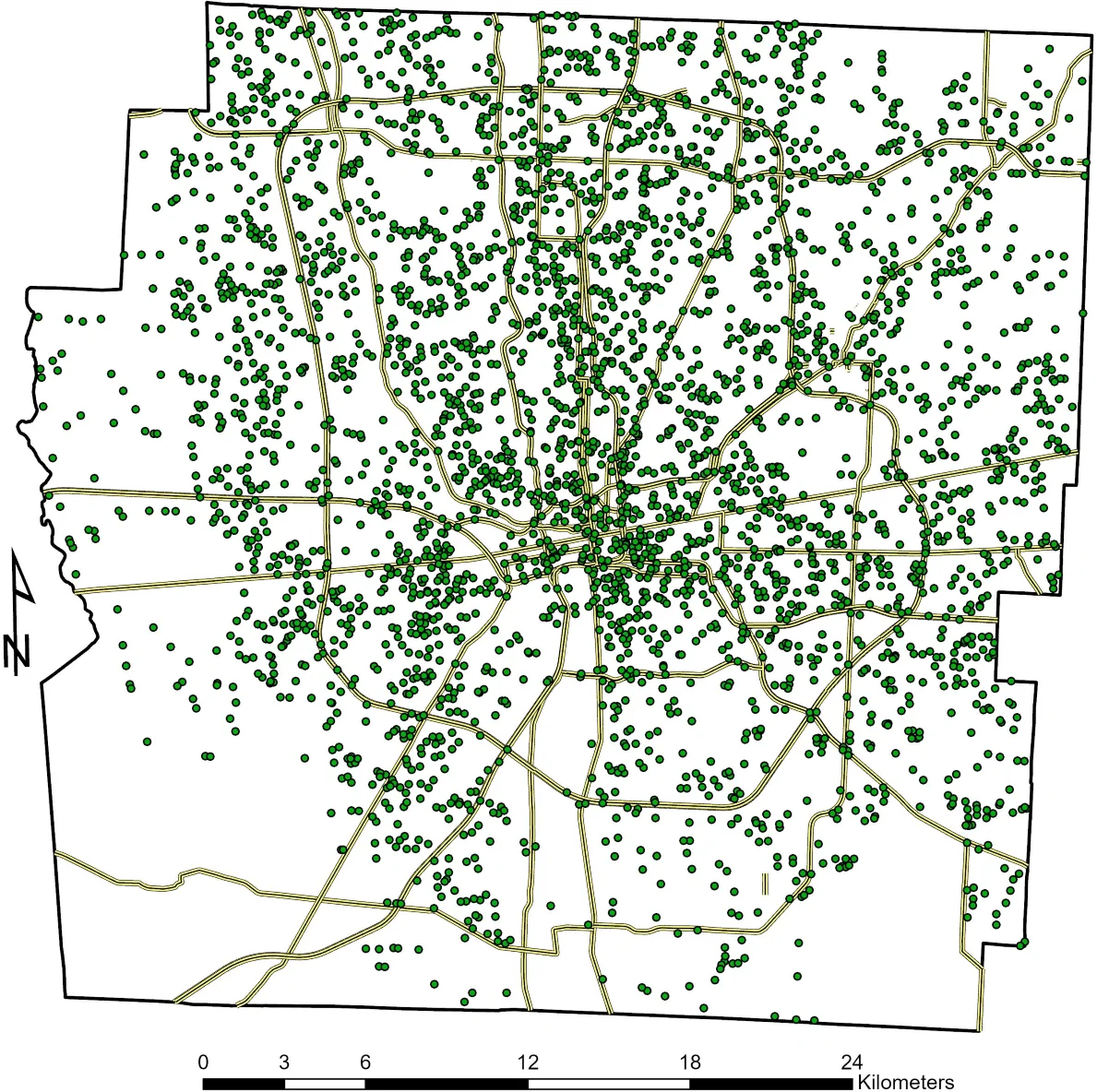
Spatial distribution of randomly selected audit location–dates throughout the study area, Franklin County, Ohio, N=8256 audits (n=2214 unique locations shown in green points).

Auditing took place at The Ohio State University Comprehensive Cancer Center between August 2022 and April 2023 using 2 computer displays, one of which was 24″ with 1920 ×1080 resolution for viewing GSV scenes. A total of 8256 audits were completed, 5751 of which met eligibility for this spatiotemporal analysis across 2214 unique locations (Figure 1). Eligibility criteria were (1) appropriate streetscape (the imagery was not of highway, a backyard, or a nature trail, and was not within a building) (n=7784, 94.3% of audits), and (2) at least 2 different dates at the location (n=5751, 70% of audits, 62.8% of locations). Among the 2214 eligible audit locations, the percentage with 3 or more unique dates was 59.7%, and the percentage with 4 or more unique dates was 14.6%. Only 1.9% of locations had 5 unique dates.

CRC survival data used in external validation analyses were from the Ohio Cancer Incidence Surveillance System. Eligible cases were adults (aged 20 years and older), diagnosed from 2012 to 2019 with an invasive cancer of the colon or rectum (*International Classification of Diseases* codes *C18.0-C18.9*, *C19.9*, *C20.9*, and *C26.0*), vital status follow-up through December 31, 2020, and geocoded residential address at diagnosis in Franklin County, Ohio. Survival time was calculated as days from date of diagnosis to end of follow-up, where end of follow-up was defined as date of death, date of last contact, or December 31, 2020. Follow-up is passive in that cases are presumed alive unless a death is found in linkages with Ohio Vital Statistics, Social Security Death Index, or the National Death Index. Covariates also from Ohio Cancer Incidence Surveillance System included age at diagnosis and stage at diagnosis (localized, regional, and distant or metastatic), sex (male or female), race-ethnicity (non-Hispanic White, non-Hispanic Black, non-Hispanic Asian, Pacific Islander, American Indian or Alaskan Native, or other; Hispanic), marital status (married and marriage-like vs widowed or divorced or separated or never married), and type of insurance (private or commercial, Medicare, Medicaid, uninsured, other).

### Neighborhood Disinvestment Score

**Figure 2.**
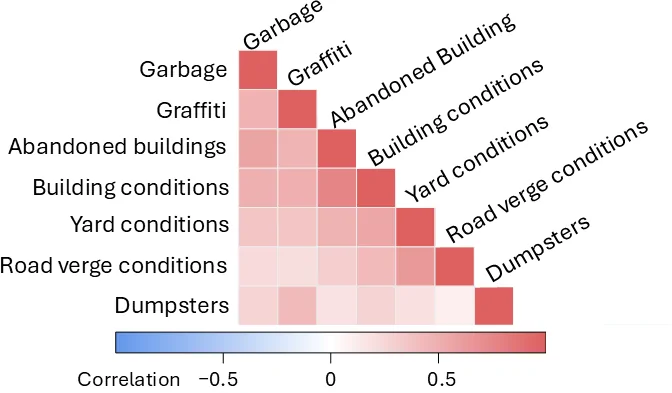
Correlations among 7 audit items related to neighborhood disinvestment, audited throughout Franklin County, Ohio, N= 5751 (2009-2022).

An item response theory analysis of responses to the 7 audit items was used to investigate item correlation structure (Figure 2), internal consistency reliability per area under the curve of the Item Response Theory model (0.955) [24], and generate an NDS [1,25].

To reduce physical disorder measurement error, we generated rater-adjusted scores by regressing physical disorder on the rater variable with 6 nominal levels (eg, 5 dummy variables treated as fixed effects). The residuals of the resulting model, which represents NDS adjusted for rater bias, were used in subsequent analysis. Throughout, we simply use NDS to refer to the rater-adjusted residuals of raw NDS.

### Spatial Cluster

**Figure 3.**
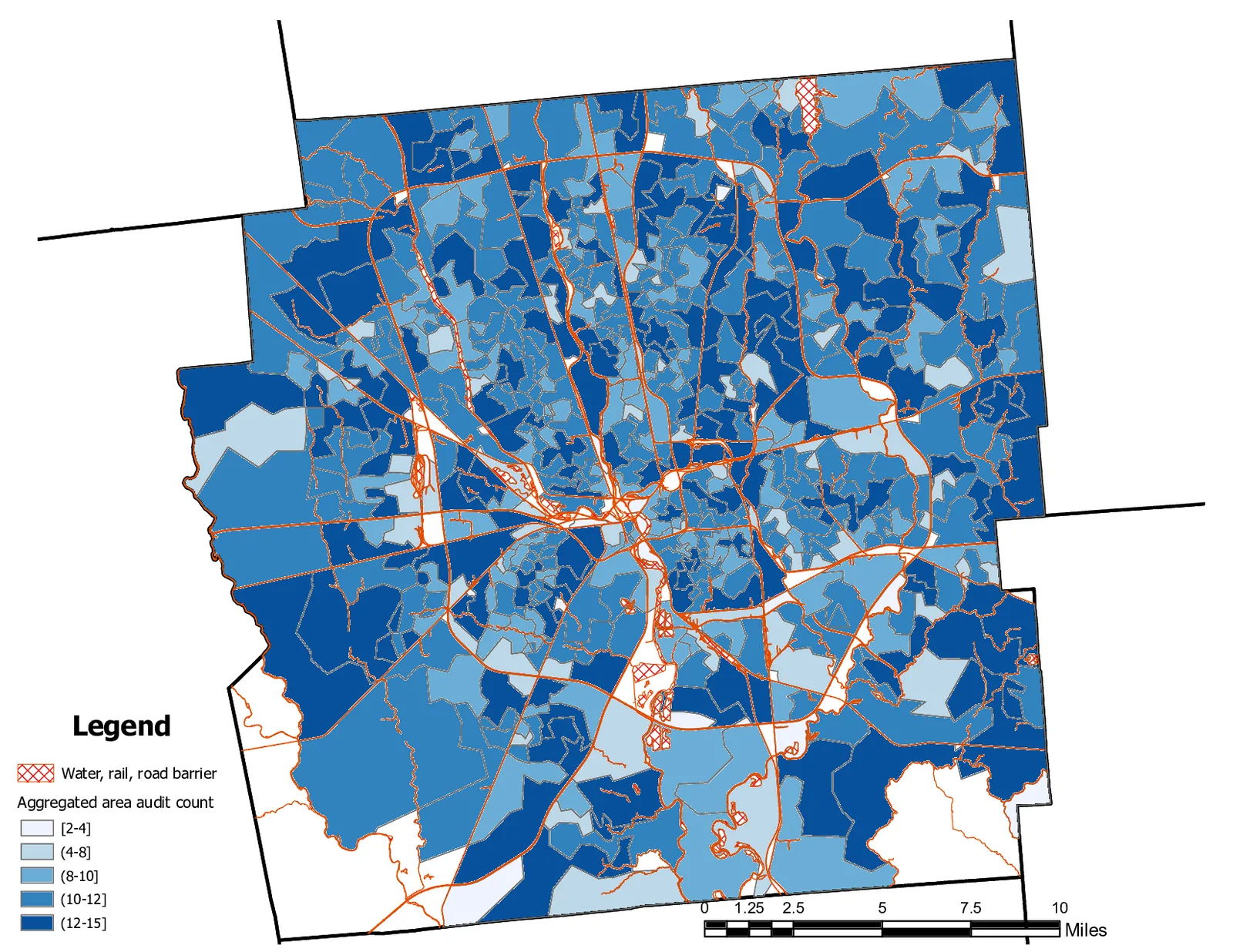
Spatial clusters colored by audit sample frequency, Franklin County, Ohio, N=5751. Darker blue indicates more observations. Physical barriers are in orange.

Previous studies have reported that social stratification such as racial-ethnic segregation can be reinforced by such physical boundaries as major roads, railroads, and bodies of water [26,27]. To test this with NDS, we partitioned the study region into mutually exclusive areas with boundaries defined by major physical barriers and then aggregated those areas based on audit samples. We assessed highways, railways, and hydrography from 2010 TIGER (Topologically Integrated Geographic Encoding and Referencing) shapefiles for Franklin County, Ohio [28]. Buffers on either side of highway and railway line files were created as estimates of typical widths (30 ft) and easements (25 ft), respectively. Polygonal hydrography was processed to estimate those features acting as barriers with potential to separate communities from one another (eg, major rivers or large lakes). Through a series of operations, hydrography features with an average width of 10 ft and length of 1 mile were considered barriers and merged with the polygonal highway and railway files to yield the physical barrier file. Next, Voronoi polygons around unique audit locations (Figure 1) were generated and then split by the intersection of physical barriers. Through a series of operations, intact and split Voronoi polygons were aggregated based on proximity to one another and final audit sample frequency to achieve areas with a maximum of 15 audits and as few as 2 (median 10). We aggregated using the Geographic Aggregation Tool implemented in R (R Core Team) [29]. The resulting spatial clusters, together with physical barriers, are shown in Figure 3. Clusters are colored by sample frequency, with barriers shown in orange. In total, there are 530 clusters.

### Spatiotemporal Modeling

We were interested in accurately predicting the NDS, denoted as *Y*(*s*_0_,*t*_0_), at a given location *s*_0_ and time *t*_0_. To effectively capture the spatial and temporal dependencies among observations, we used a spatiotemporal model estimating NDS that accounts for correlations across both space and time:


(1) $$Y\left(s_{i},t_{j}\right)\text{=}\mu \left(s_{i},t_{j}\right)\text{+}\epsilon \left(s_{i},t_{j}\right),$$

where *µ*(*s_i_*,*t_j_*) is the population mean trend and *ε*(*s_i_*,*t_j_*) is a zero-mean residual process that captures spatiotemporal dependencies through a specified covariance structure. The specific choice of the covariance structure will be discussed in “Residual Process” section.

#### Mean Trend

A well-specified *µ*(*s_i_*,*t_j_*) accounts for large-scale variation by modeling the spatial and temporal effect of NDS using available information. A common approach is to model the mean trend using linear regression, incorporating covariates that capture these effects. We consider 2 mean trend models as candidates, with covariate information detailed in the rest of this section. We compare model fit and predictive performance in the “Model Comparison” section.

*Model 1*: A fixed effect model including polynomials of precise spatial location (longitude and latitude), year, and seasonality effect.


(2) $$\begin{array}{l} \mu (s_{i},t_{j})=\beta_{0}+\beta_{1}\;\mathrm{longitude}(s_{i})+\beta_{2}\;{\mathrm{longitude}}^{2}(s_{i})\\ +\beta_{3}\;\mathrm{latitude}(s_{i})+\beta_{4}\;{\mathrm{latitude}}^{2}(s_{i})+\beta_{5}\;\mathrm{longitude}\\ \left(s_{i})\times \mathrm{latitude}(s_{i})+\beta_{6}\;\mathrm{season}(t_{j})+\beta_{7}\;\mathrm{year}(t_{j}\right) \end{array}$$

*Model 2-R*: A mixed effect model including spatial cluster as random intercept. Year and seasonality are included as fixed effects.


(3) $$\mu \left(s_{i},t_{j}|\gamma_{k}\right)\text{=}\beta_{0}\text{+}\beta_{1}season\left(t_{j}\right)\text{+}\beta_{2}year\left(t_{j}\right)\text{+}\gamma_{k}.$$

1. longitude (*s*_*i*_) and latitude (*s*_*i*_) represent *precise spatial information* at location *s*_*i*_. Spatial information in model 1 (equation 2) is captured using second-order polynomials of longitude and latitude and their first-order interaction.
2. season (*t*_*j*_) represents *seasonal effect* at time *t*_*j*_. It represents temporal variation within each year. We model seasonality as a categorical variable with 4 levels: January-March (season 1, winter), April-June (season 2, spring), July-September (season 3, summer), and October-December (season 4, fall). The last level (season 4, fall) serves as the reference level. Thus, the model coefficients for season form a 3-dimensional vector representing the effects of the remaining 3 seasons (season 1, 2, and 3) compared with season 4.
3. year (*t*_*j*_) represents *large-trend temporal effect* at time *t*_*j*_. It is modeled as the number of years since the earliest audit sample. For instance, the image date of the earliest audit sample is March 1, 2009, which corresponds to year=1. Correspondingly, samples from 2010 have year (*t*_*j*_) = 2, samples from 2011 have year (*t*_*j*_) = 3, and so on. The corresponding model coefficients for year are scalars, capturing the overall temporal trend over the years.
4. *γ_k_* represents the random effect for the *k*th *spatial cluster* for *k* ∈ {1,...,530}, where *k* indicates which spatial cluster location *s_i_* belongs to in Figure 3. We assume *γ_k_ ∼ N* (0, *σ_γ_*). Using a cluster can be viewed as modeling a coarser spatial effect compared with precise location information as in model 1. Nevertheless, incorporating the spatial cluster effect allows for nonsmooth local variation. Geographically adjacent regions may exhibit significantly different NDS, and covariance and spillover may be affected by physical barriers (eg, NDS may be different on the “wrong side of the tracks”). A random cluster effect can account for between-cluster variability while borrowing information across clusters.

To summarize, model 1 included precise geographic information through longitude and latitude polynomials that may lead to more precise NDS prediction, while model 2-R accounted for physical barriers through spatial cluster random intercepts and allowing for nonsmooth NDS changes, which can mimic real-world settings more closely [30,31]. In addition to model 1 and model 2-R, we also include and compare a fixed effect spatial cluster model (called *model 2-F*) in Multimedia Appendix 1.

#### Residual Process

The zero-mean residual process *ε*(*s_i_,t_j_*) in equation 1 captures the remaining spatiotemporal dependencies among observations that are not explained by the mean trend. Assuming the process is second-order stationary and isotropic, we model the covariance between 2 observations, *Y*(*s*, *t*) and *Y*(*s′*, *t′*), using a simplified sum-metric covariance structure [32-35]. The simplified sum-metric covariance structure was selected for 3 main reasons. First, it is more flexible than separable models, which assume that the spatial correlation pattern does not change over time. This assumption may not hold for neighborhood disinvestment, which can exhibit different temporal patterns across locations. Second, unlike the metric model that has only a single covariance function, it does not require space and time to be “comparable,” which is appropriate given their fundamentally different units (kilometers vs days). Third, compared with fully nonseparable models, the sum-metric structure is more interpretable: it decomposes the total covariance into spatial, temporal, and joint components while keeping the number of parameters manageable for estimation. In addition, comparing with a sum-metric covariance, the word “simplified” refers to the addition of a separate *nugget effect* at the overall level, which captures measurement error and microscale variation occurring at distances smaller than the resolution of the sampling design.

Specifically, the simplified sum-metric covariance function is given by

,(4) $$\begin{array}{l} Cov\left(Y\left(s,t\right),Y\left(s^{\prime },t^{\prime }\right)\right)\text{=}Cov\left(\epsilon \left(s,t\right),\epsilon \left(s^{\prime },t^{\prime }\right)\right)\text{=}C_{s}\left(d_{s}\left(s,s^{\prime }\right)\right)\text{+}\\ C_{t}\left(d_{t}\left(t,t^{\prime }\right)\right)\text{+}C_{st}\left(d_{st}\left(\left(s,t\right),\left(s^{\prime },t^{\prime }\right)\right)\right)\text{+}\tau^{2}I\left\{s\text{=}s^{\prime },t\text{=}t^{\prime }\right\} \end{array}$$

where *C_s_*, *C_t_* and *C*_*st*_ represent the spatial, temporal, and joint spatiotemporal covariance functions, respectively, *I* {⋅} is the indicator function, which equals 1 when *s*=*s*', *t*=*t*', and 0 otherwise. The distances are defined as follows:

- Spatial distance: $d_{s}\left(s,s^{`}\right)\text{=}\sqrt{{\left(x\left(s\right)\text{-}x\left(s^{`}\right)\right)}^{2}\text{+}{\left(y\left(s\right)\text{-}y\left(s^{`}\right)\right)}^{2}}$, measured in kilometers.
  *x* (*s*) and *y* (*s*) are the coordinates at audit sample location *s* based on projected equidistant conic.
- Temporal distance: $d_{t}\left(t,t^{`}\right)\text{=}\text{|}t\text{-}t`\text{|}$, measured in days.
- Joint spatiotemporal distance: $d_{st}\left(\left(s,t\right),\left(s^{\prime },t^{\prime }\right)\right)\text{=}\sqrt{d_{s}{\left(s,s^{\prime }\right)}^{2}\text{+}stAni\cdot d_{t}{\left(t,t^{\prime }\right)}^{2}}$. Here, *stAni* is the *spatiotemporal anisotropy* parameter, which rescales the relative contribution of the temporal distance to the joint spatiotemporal distance.

In addition, the last term *τ*^2^ is the nugget effect, which represents the variance of observation error, occurring when both *d_s_* (*s*, *s*') = 0 and *d_t_* (*t*, *t*') = 0 .

Each covariance function *C_s_*, *C_t_* and *C_st_* is modeled using the spherical exponential covariance function. It has the following form:


(5) $$C(d)=\left\{ \begin{array}{ll} \sigma^{2}\left(1-\frac{3}{2}\cdot \frac{d}{\phi }+\frac{1}{2}{\left(\frac{d}{\phi }\right)}^{3}\right), & \text{if }d\leq \phi ,\\ 0, & \text{if }d§gt;\phi , \end{array}\right.$$

where *σ*^2^ and φ are the partial sill and range parameters, respectively. Combining the 3 covariance functions with equation 4, the full covariance model for *ε*(*s, t*) has 8 parameters: spatiotemporal anisotropy *stAni*; nugget effect *τ*^2^; spatial, temporal, and joint partial sills σ_*s*_^2^, σ_*t*_^2^, σ_*st*_^2^; and spatial, temporal, and joint ranges *φ_s_*, *φ_t_*, *φ_st_*. We used a *semivariogram* to estimate the covariance model in equation 4, including visually assessing its empirical version to confirm fit. The semivariogram is defined as


(6) $$\gamma \left(h,u\right)\text{=}\frac{1}{2}Var\left[Y\left(s,t\right)\text{-}Y\left(s^{\prime },t^{\prime }\right)\right]\text{=}:C\left(0,0\right)\text{-}C\left(h,u\right),$$

where *h*=*d*_*s*_(*s*, *s*') is the spatial lag, *u*=*d_t_*(*t*, *t*') is the temporal lag, and *C*(*h*, *u*) is the covariance function defined in equation 4 at spatial lag *h* and temporal lag *u*. The empirical (binned) semivariogram is the sample version of equation 6 calculated from the data. Specifically, given *K*_1_ spatial bins $I_{k_{1}}^{s}\text{=}(h_{k_{1}\text{-}1},h_{k_{1}}]$ for $k_{1}\text{=}1,\ldots ,K_{1}$ and $K_{2}$ temporal bins $I_{k_{2}}^{t}\text{=}(u_{k_{2}\text{-}1},u_{k_{2}}]$ for $k_{2}\text{=}1,\ldots ,K_{2}$, the midpoint of each spatiotemporal bin is $h_{k_{1}}^{m}\text{=}\frac{h_{k_{1}}\text{-}h_{k_{1}\text{-}1}}{2}$, $u_{k_{2}}^{m}\text{=}\frac{u_{k_{2}}\text{-}u_{k_{2}\text{-}1}}{2}$. In total, there are *K*_1_*K*_2_ midpoints. For each spatiotemporal bin with midpoint $\left(h_{k_{1}}^{m},u_{k_{2}}^{m}\right)$, the empirical semivariogram is given by:


(7) $$\hat{\gamma }\;\left(h_{k_{1}}^{m},\;u_{k_{2}}^{m}\right)=\frac{1}{2\left|N\;\left(h_{k_{1}}^{m},\;u_{k_{2}}^{m}\right)\right|}\sum_{\left\{(s_{i},t_{j}),(s_{i^{\prime }},t_{j^{\prime }})\}\in N(h_{k_{1}}^{m},\;u_{k_{2}}^{m}\right)}{\left(Y(s_{i},t_{j})-Y(s_{i^{\prime }},t_{j^{\prime }})\right)}^{2}$$

where the set of observations in each bin is:


$$N\left(h_{k_{1}}^{m},u_{k_{2}}^{m}\right)\text{=}\left\{\left\{\left(s_{i},t_{j}\right),\left(s_{i^{\prime }},t_{j^{\prime }}\right)\right\}:d_{s}\left(s_{i},s_{i^{\prime }}\right)\in I_{k_{1}}^{s},d_{t}\left(t_{j},t_{j^{\prime }}\right)\in I_{k_{2}}^{t}\right\},$$

where *k*_1_ =1,...,*K*_1_, *k*_2_ =1,...,*K*_2_, and $\text{|}N(h_{k_{1}}^{m},u_{k_{2}}^{m})\text{|}$ is the number of pairs of observations. In practice, *K*_1_ and *K*_2_ are determined by user-specified bin widths or a chosen number of spatial and temporal lags. These choices are guided by exploratory data analysis to ensure that each bin contains enough pairs of observations for stable estimation. Based on the theoretical and empirical semivariograms in equation 6 and equation 7, we can estimate the 8 covariance parameters using least squares. The results are presented in “Covariance Structure Fit” section.

#### Spatiotemporal Universal Kriging

Using this spatiotemporal model, we can predict NDS at any new (location and time) point (*s*_0_, *t*_0_) within a spatiotemporal domain defined by *s*_0_ falling in Franklin County and *t*_0_ in 2009‐2020. The prediction is given by:


(8) $$\hat{Y}(s_{0},t_{0})=x_{0}^{T}\hat{\beta }+{\hat{A}}^{T}{\hat{\Sigma }}^{-1}\left(Y-X\hat{\beta }\right)$$

where

- ***x***_0_ is the vector of covariates, that is, season, year, precise location polynomials, or spatial cluster at location *s*_0_ and time *t*_0_.
- *$\hat{\beta }$*is the estimated coefficient vector for mean trend model 1, 2-F or 2-R.
- ***X*** is the design matrix for *n* observed samples.
- $Y\text{=}{\left(Y\left(s_{1},t_{1}\right),\ldots ,Y\left(s_{n},t_{n}\right)\right)}^{T}$ is the vector of observed NDS.
- $\hat{\Sigma }$ is the *n*×*n* estimated sample covariance matrix, obtained using the fitted covariance model.
- *$\hat{A}$*is the n-dimensional estimated covariance vector between the observed samples and the new (location and time) point.

The first term *x*_0_^*T_$\hat{\beta }$_*^ corresponds to the mean trend prediction, which is based on observable factors such as season, year, and precise location or spatial cluster. Furthermore, the second term ${\hat{A}}^{T}{\hat{\Sigma }}^{-1}\left(Y-X\hat{\beta }\right)$ adjusts the mean trend prediction by using the covariance structure to borrow information from neighboring (location and time) points in the observed data. Compared with mean trend prediction *$\hat{Y}$*(*s*_0_, *t*_0_) = *x*_0_^*T_$\hat{\beta }$_*^, which assumes independence among observations, universal Kriging is expected to provide more accurate predictions for data with spatiotemporal dependencies. We compare the accuracy of our mean trend and Kriging predictions in “Out-of-Sample Prediction Accuracy” section, where we use a cross-validation–based method to calculate the mean prediction error at specific audit locations and times using a subset of the original 5751 audit samples.

### Exposure Time Lag Validation

We used this spatiotemporal universal Kriging model to estimate averaged, monthly values of disorder at set time lags and durations from CRC diagnosis date. The spatial points used for Kriging are exact geocoded latitude and longitude of patients’ residential address at diagnosis. We then used these values to test associations with CRC accelerated failure time models using a Weibull distributional assumption on the survival time and adjusted for covariates. A priori times for estimating physical disorder were the exact date of diagnosis and prediagnosis monthly averages at 3-month intervals up to 2 years prediagnosis (0‐3 months, 0-6 months, 0-9 months,..., 0‐24 months).

Model covariates were age, sex, marital status, minoritized race-ethnicity, health insurance, stage at diagnosis, and the interaction between stage at diagnosis and physical disorder. Based on previous studies, we hypothesized a stage-neighborhood disinvestment interaction wherein survival time among those diagnosed at stage 2, but not stages 1 and 3, would be shorter for those living in disinvested neighborhoods [1].

### Ethical Considerations

The Ohio State University Institutional Review Board (IRB) approved this study (IRB number 2023C0088, most recent approval: September 3, 2025). The IRB approved a waiver of consent, as such no participant contact nor compensation for research participation was provided. Identifiable data were geocoded residential address and dates of diagnosis of cancer cases, which were analyzed only on encrypted, password-protected computers and by IRB-approved study personnel who completed all required responsible conduct of research training.

All analyses in “Results” section were implemented using the statistical computing software R (R Core Team) [36]. Specifically, the spatiotemporal model fitting and universal Kriging predictions were performed primarily using the gstat package [33,34]. The accelerated failure time model in exposure validation analysis is fitted using R package survival [37]. Reproducible code and the entire audit protocol training manual are provided in the supplementary materials.

## Results

### Exploratory Data Analysis

Overall, 62.8% of locations had at least 2 available dates. Among these eligible locations, only 1.9% had 5 available dates that approximated a uniform temporal distribution important for assessing longitudinal trends (ie, dates available for each of: earliest 2009+, most recent, middate, and approximations of the 25th and 75th percentiles between earliest and most recent). However, 59.7% of locations had at least 3 unique dates available. The spatial distribution of audits representing the midpoint and 25th or 75th percentiles appear less dense toward the county periphery and away from the City of Columbus compared with distributions of the earliest or most recent images that cover all but the Southwest corner of the county (Figure S1 in Multimedia Appendix 1). Given the temporally uniform sampling protocol and audit dates (2022‐2023), the distribution of years by target date indicated complementary and some overlapping distributions of image years; 54% of “earliest” audits were 2009, 94% of “25th percentile” audits were between 2011 and 2015, 89% of “midpoint” audits were between 2014 and 2017, 59% of “75th percentile” audits were between 2018 and 2020, and 56% of “most recent” audits were 2021 or 2022. Wintertime images were rarer and 86% were taken between May and October.

**Figure 4.**
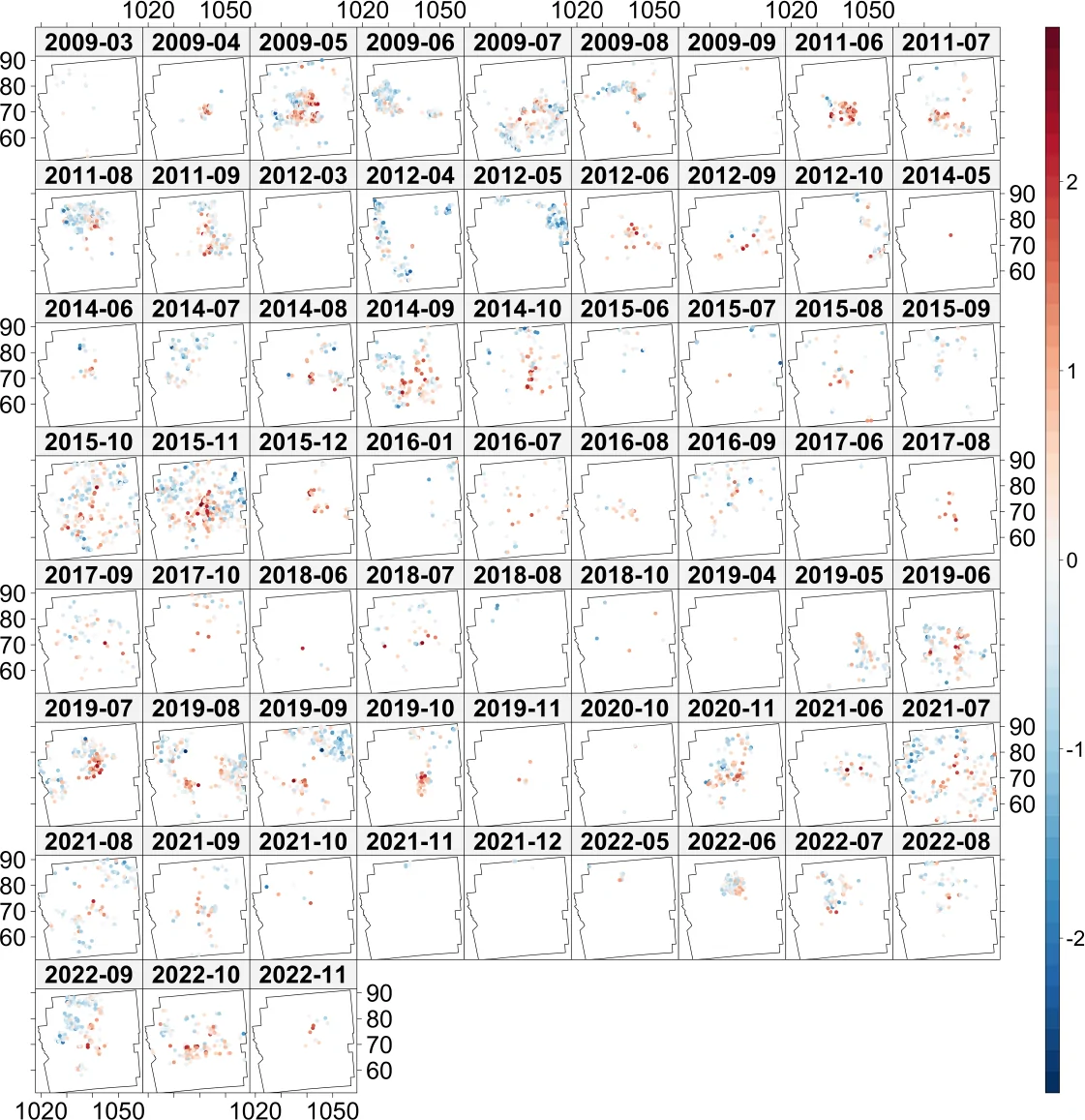
Observations of neighborhood disinvestment score at different locations and time in Franklin County, Ohio. N=5751, 2009-2022.

Figure 4 shows the spatial and temporal distribution of NDS. Overall, the NDS ranges from −2.818 to 2.816, with mean −0.002, median −0.057, SD 0.809, skewness 0.225, and kurtosis 3.166. The distribution is roughly symmetric with no strong outliers. Each panel corresponds to an observation date (the first day of the month) and each point within a panel represents a location. Points are colored by values of NDS, with a gradient from blue through white to red. Notably, certain dates, such as 2017‐06 and 2019‐04, have only 1 data point. Also, for specific dates, such as 2009‐05 and 2014‐09, the spatial distribution of observations is unevenly dispersed, with central areas more extensively sampled and rural areas less frequently observed. These underscore the sparse and irregular structure of the observations of NDS and motivate the necessity for the modeling technique we chose, which can accommodate sparse and irregular spatiotemporal data. Additionally, spatial correlation patterns are noticeable within each panel, with nearby locations exhibiting similar NDS. In general, urban neighborhoods have higher NDS than rural areas. To quantitatively evaluate spatiotemporal autocorrelation, we compute a spatiotemporal Moran I [38], where we assign weight 1 to “spatiotemporal” neighbor defined based on the estimated spatial and temporal range in Table 1, which is generated based on the method described in the “Residual Process” section and will be discussed in the “Out-of-Sample Prediction Accuracy” section. The spatiotemporal Moran I for NDS is 0.320, with *P* value <.001 for a permutation test that randomly permutes NDS 1000 times and recalculates Moran I. This suggests that there exists positive spatiotemporal autocorrelation for NDS.

**Table 1.** Estimated parameters of simplified sum-metric covariance model.

Parameter	st*Ani*	σ_s_^2^	σ_*t*_^2^	σ_*st*_^2^
Estimate	0.056	0.238	0.038	0.052
Parameter	*τ* ^2^	φ_*s*_	φ_*t*_	φ_*st*_
Estimate	0.268	2.769	270.000	0.005

### Model Comparison

We compare and evaluate the 2 mean trend models, model 1 and model 2-R, from 2 perspectives: in-sample model fit and out-of-sample prediction accuracy. The corresponding results for model 2-F are shown in Multimedia Appendix 1.

#### In-Sample Mean Trend Model Fit

We use all the samples in the data, fit the mean trend models, and compare their Akaike information criterion (AIC) and Bayesian information criterion (BIC). Both AIC and BIC evaluate model fit based on likelihood and complexity, with BIC having more penalization on complexity (number of parameters). A smaller AIC or BIC suggests a better model fit. Moreover, we additionally compare the 2 models with their corresponding reduced version that excludes season as a covariate. We denote the reduced version of model 1 and model 2-R as model 1r and model 2r-R, respectively.

Table 2 shows the AIC and BIC of the 4 candidate models, with the smallest value in italic. For both pairs of models, the full model has a better fit, that is, a smaller AIC and BIC, than the reduced version, suggesting that season should be included as one of the covariates. Model 2-R has smaller AIC and BIC than model 1, suggesting that it has better in-sample fit based on these criteria. Nonetheless, to assess overfitting, we further evaluate out-of-sample prediction accuracy for the 2 full models in the “Out-of-Sample Prediction Accuracy” section.

**Table 2.** AIC^a^ and BIC^b^ for candidate models^c^.

Models	AIC	BIC
Model 1	12951.320	13024.548
Model 1r	12987.415	13040.672
Model 2-R	*12183.205*	*12229.805*
Model 2r-R	12214.282	12240.911

a AIC: Akaike information criterion.

b BIC: Bayesian information criterion.

c R stands for spatial cluster random intercept model and r stands for reduced models without seasonal effect.

Table 3 shows the estimated coefficients, their corresponding test statistics (*t* value), and *P* values of the fixed effect covariates shared between 2 models, that is, season and time. The coefficients have same directions, similar magnitude, and *P* values for both models. Compared with season 4 (fall), season 2 (spring) and season 3 (summer) have significantly lower NDS, meaning on average there are lower visual indications of neighborhood disinvestment in spring and summer than in fall. Time effect (integer year) is positive and significant. Nonetheless, due to the small magnitudes of the coefficients, the time effect does not lead to large changes in NDS within the time span of the data. For example, for model 1, an increase of 14 years on average results in an increase of NDS of 0.01×14=0.14. This magnitude is similar to seasonal coefficients. For model 1, *P* values for the first- and second-order latitude and longitude polynomials are <.001, and *P* value=.04 for the first-order interaction between longitude and latitude. This suggests that location information serves as strong predictors for NDS.

**Table 3.** Coefficients, test statistics, and *P* values for season and time fixed effects.

Fixed effect	Model 1			Model 2-R		
	Coefficient	*t* test (*df*)	*P* value	Coefficient	*t* test (*df*)	*P* value
Season 1	0.139	1.159 (5741)	.25	−0.050	−0.459 (5217)	.65
Season 2	−0.154	−5.115 (5741)	<.001	−0.162	−5.948 (5217)	<.001
Season 3	−0.147	−5.779 (5741)	<.001	−0.105	−4.544 (5217)	<.001
Time	0.010	4.248 (5741)	<.001	0.005	2.747 (5217)	.006

#### Out-of-Sample Prediction Accuracy

To compare the out-of-sample Kriging prediction accuracy using the 2 mean trend models, as well as mean trend only prediction assuming independence among observations (“Residual Process” section), we split the data into training set with sample size n_1_=4300 (75% of samples) and validation set with sample size n_2_=1451. To ensure that there are enough training data within each spatial cluster, we construct the training set as follows. First, we include all samples in clusters where there are at most 5 observations, then we randomly sample 5 observations from each of the remaining clusters that have more than 5 observations. Finally, we uniformly sample from the remaining observations until the training set sample size reaches n_1_. We fit mean trend and residual covariance model (see “Covariance Structure Fit” section) on the training set and evaluate out-of-sample RMSPE on the validation set. We repeat the random split of training and validation set 30 times and calculate the mean and SE of RMSPE for each of the models. The results are shown in Table 4. Compared with mean trend only predictions, Kriging predictions have smaller RMSPE for both models. Furthermore, model 1 Kriging predictions have smaller RMSPE than model 2-R. This indicates that incorporating precise location in the mean trend model and introducing spatiotemporal dependencies in the residual process jointly yield more accurate predictions comparing with coarser spatial cluster in the mean trend (model 2-R) or assuming sample independence (mean trend only prediction). Therefore, we use model 1 as mean trend model for subsequent results.

**Table 4.** Out-of-sample RMSPE with standard error within parenthesis.

Models	Model 1	Model 2-R
Mean trend only	0.751 (0.002)	0.673 (0.002)
Kriging	0.651 (0.002)	0.662 (0.002)

To further evaluate whether the temporal component of the spatiotemporal Kriging model provides prediction gains beyond a purely spatial approach, we conduct an additional comparison using 5-fold cross-validation (repeated 10 times) under model 1. Three approaches are compared: (1) spatial-only Kriging using a single randomly selected observation per unique location (n=2222); (2) spatial Kriging with jittering, replacing the spatial coordinates of repeated measurements at the same location with unique coordinates sampled uniformly on a 10-meter radius circle centered at the original location (n=5751); and (3) the full spatiotemporal Kriging model (n=5751). Mean RMSPE and SE across folds for these 3 methods are 0.672 (SE 0.003), 0.643 (SE 0.001), and 0.634 (SE 0.001), respectively. The prediction error reduction from spatial-only to jittered spatial Kriging reflects the gain because of a larger sample size, while further reduction from jittered spatial to spatiotemporal Kriging isolates the improvement from explicitly modeling temporal autocorrelation.

To get a better understanding of the prediction performance of model 1, we aggregate the predictions over 30 training-validation splits, calculate the averaged values as the final predictions, and compute the residuals. Note that due to data sparsity in some spatial clusters, some data points do not appear in validation sets and therefore do not have a predicted value. In total, we get predictions for 98.1% (5643/5751) unique data points. Figure S1 in Multimedia Appendix 1 shows the residuals split by dates of audit samples as described in “Study Region and Audit Sample” section.

### Covariance Structure Fit

Following methods described in “Residual Process” section, we fit the empirical and theoretical semivariogram based on the simplified sum-metric covariance model on the residuals obtained from the mean trend model fit. The residuals have a sample mean 0 (SD 0.745), skewness 0.054, and kurtosis 3.110. The 1-sample Kolmogorov-Smirnov test results in a test statistics *D*=0.009 (*P* value=.75). These suggest that there is no strong evidence of deviation from normality. Table 1 shows the 8 covariance parameter estimates. Recall from “Residual Process” section, these include spatiotemporal anisotropy *stAni*, nugget effect *τ*^2^, partial sills σ_*s*_^2^, σ_*t*_^2^, σ_*st*_^2^ (spatial, temporal, and joint), and ranges φ_*s*_, φ_*t*_, φ_*st*_ (spatial, temporal, and joint). Both the spatial and temporal components contribute to the residual dependencies. The estimated spatial range $\hat{\phi }$_*s*_=2.769 suggests that spatial correlation becomes negligible beyond 2.8 kilometers. The temporal range estimate $\hat{\phi }$_*t*_=270 days suggests that temporal dependency exists in a moderate period of time, but the magnitude of the dependency, indicated by partial sill estimate $\hat{\sigma }$_*t*_^2^=0.038, is very small. The joint range and partial sill estimates, $\hat{\phi }$_*st*_=0.005 and $\hat{\sigma }$_*st*_^2^=0.052, imply that the joint effect is concentrated within a very small spatiotemporal neighborhood with small variance. The nugget effect estimate *$\hat{\tau }$*^2^=0.268 suggests the measurement error and the amount of unexplained variance are relatively large. In the context of neighborhood disinvestment, such variability may arise from differences in auditing image quality or local features not captured by the broader spatial or temporal trends. Finally, a small spatiotemporal anisotropy estimate *$\hat{stAni}$*=0.056 indicates that temporal distance has much smaller contribution to the joint covariance than spatial distance.

**Figure 5.**
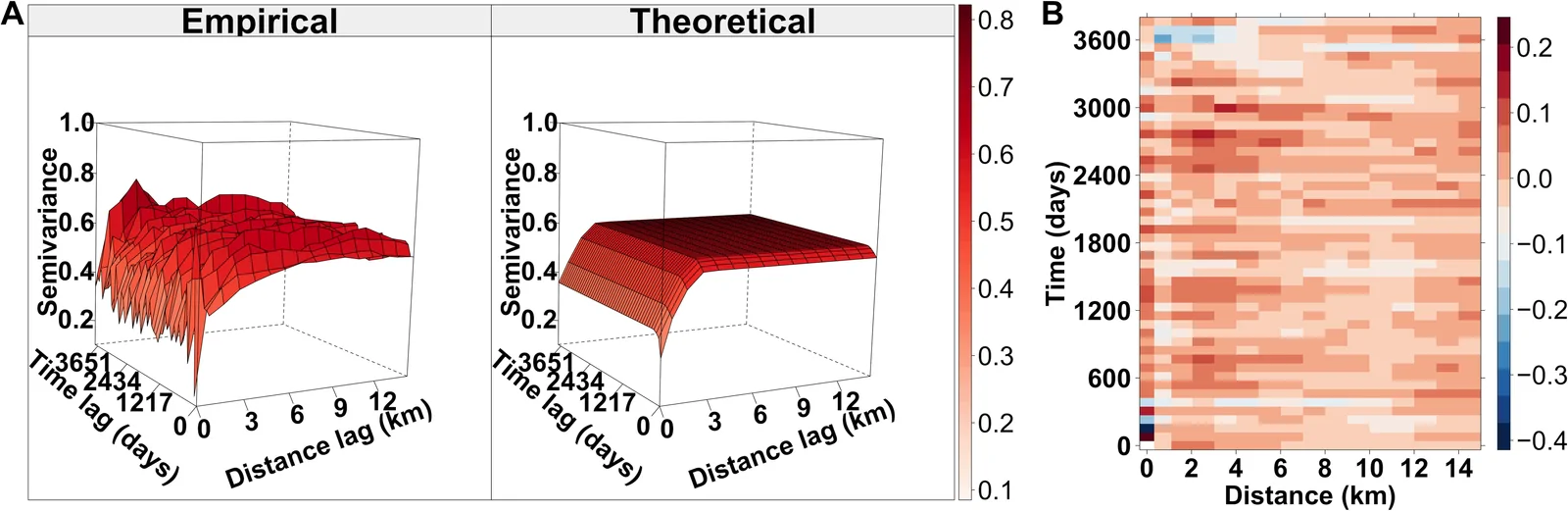
(A) Left: empirical semivariogram fit. Right: theoretical semivariogram fit. (B) Residuals of theoretical semivariogram fit.

Figure 5A shows the empirical versus theoretical semivariogram fit in 3D, and Figure 5B shows the residual of the theoretical semivariogram fit in 2D. The trends of the theoretical semivariogram in Figure 5A match the parameter estimates, for example, a moderate spatial range and a weak temporal dependency. From Figure 5B, the absolute residuals are larger when the spatiotemporal distance is small. This may be due to unmodeled fine-scale variation and measurement noise, which are more pronounced at short ranges and not fully captured by the smooth structure of the simplified sum-metric model.

#### Kriging Prediction

**Figure 6.**
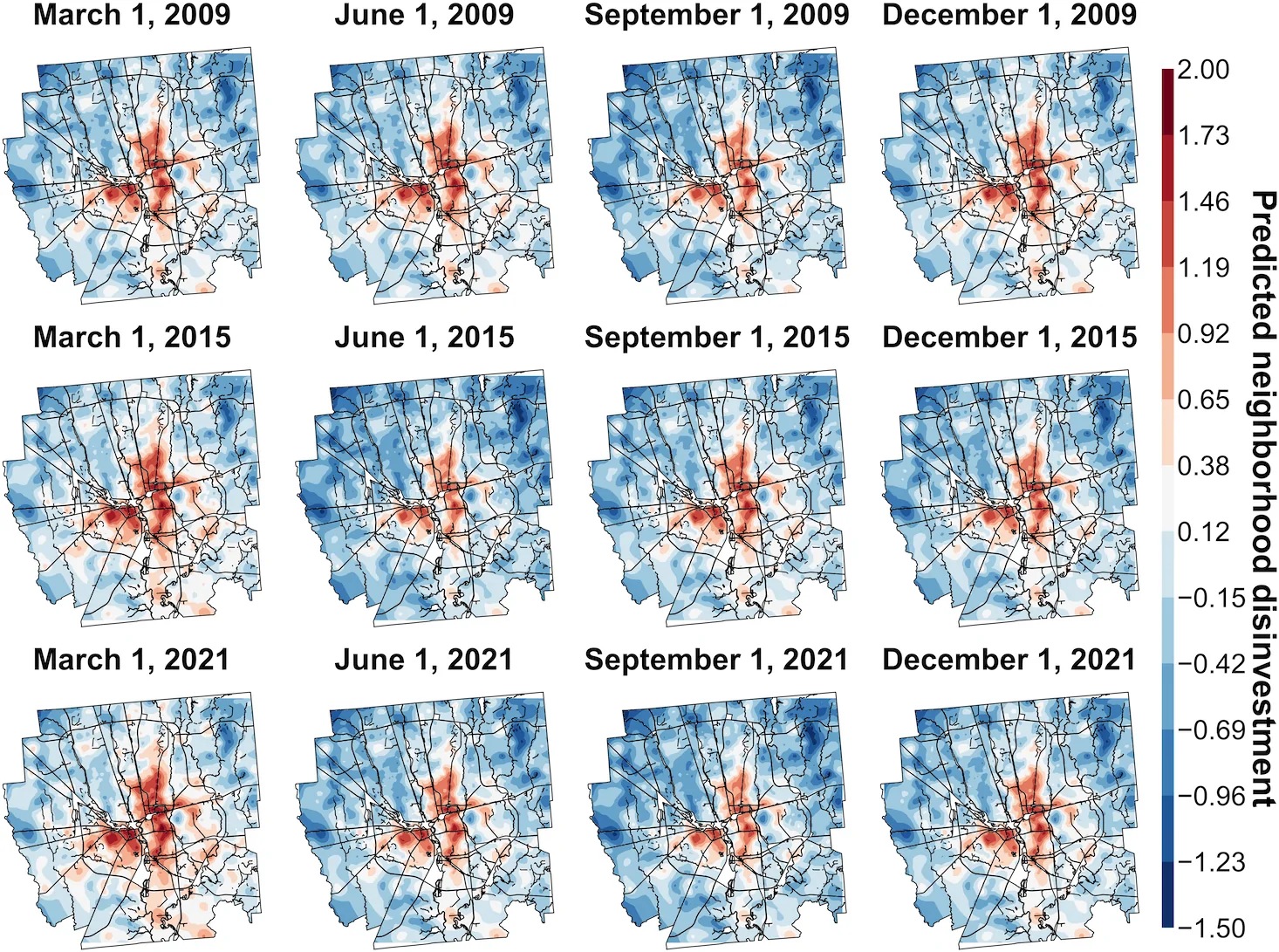
Kriging predictions of neighborhood disinvestment score with 0.1-kilometer precision for 4 seasons in 2009, 2015, and 2021 in Franklin County, Ohio.

Figure 6 shows the Kriging prediction of NDS for different seasons (March 1, June 1, September 1, and December 1) in 2009, 2015, and 2021. In each panel, Kriging is performed on a 0.1-kilometer grid of spatial points across Franklin County at the exact time points shown in the subtitles. The spatial trend is similar within each panel and coincides with the observations in Figure 4: urban areas have higher NDS than rural areas. Overall, the predictions exhibit very small temporal trends from 2009 to 2021 by a visual comparison within each column. On the other hand, the seasonal effects are more evident: summer (June 1) has much lower NDS than winter (March 1), indicated by lighter red regions in urban areas and darker blue regions in rural areas when comparing column 2 with column 1 within each row. This matches the estimated seasonal effects in the mean trend model as shown in Table 3.

#### Exposure Validation on CRC Survival

We use Kriging predictions of NDS to investigate the association between disinvestment exposure and CRC survival. The dataset includes 2727 patients with an average age of 62.705 years. Demographically, 48.8% (1331/2727) are male and 51.2% (1396/2727) are female, 72.2% (1969/2727) are non-Hispanic White, and 44.4% (1210/2727) are married or have a partner. For types of health care insurance, 42.8% (1167/2727) have Medicare, 33.6% (917/2727) have private insurance, 10.1% (275/2727) have Medicaid, 3.0% (83/2727) have other insurers, and 4.2% (115/2727) are uninsured. For tumor stages at diagnosis, 29.4% (803/2727) were diagnosed at stage 1 (localized), 40.8% (1113/2727) at stage 2 (regional), and 21.5% (587/2727) at stage 3 (distant). In total, there were 724 CRC deaths within our follow-up period. The median follow-up time per patient is 36.581 months. There are 13.2% (359/2727) of observations with at least 1 missing value in the predictors, and we include samples only without any missingness in the survival modeling (n=2368).

**Figure 7.**
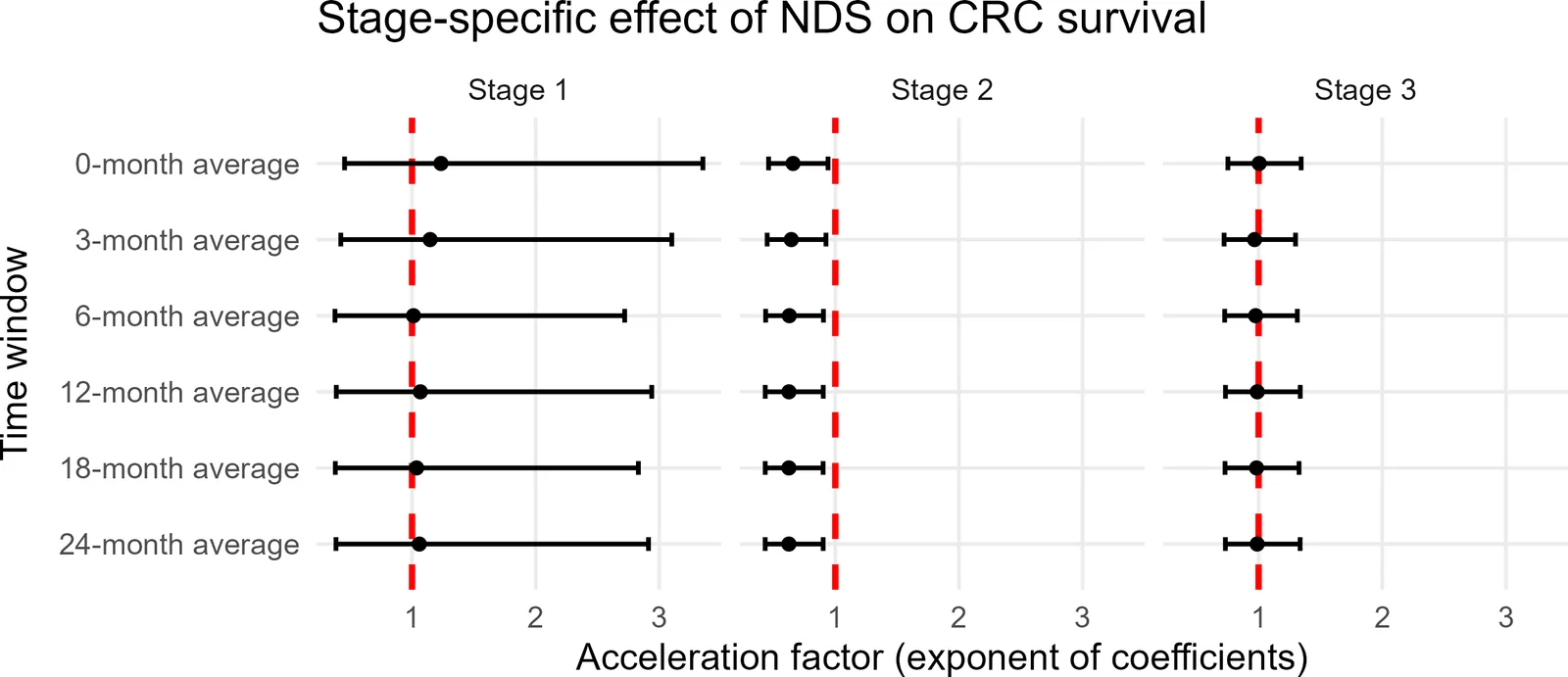
Estimated stage-specific acceleration factors (exponent of coefficients) of neighborhood disinvestment score averaged over 0, 3, 6, 12, 18, and 24 months prior to diagnosis on CRC survival (dots) and their 95% CIs (horizontal bars). Left to right: stages 1-3. CRC: colorectal cancer; NDS: neighborhood disinvestment score.

As described in “Exposure Time Lag Validation” section, NDS averaged over 0, 3, 6, 12, 18, and 24 months prior to diagnosis was included as a covariate in each survival model. The NDS values are obtained by Kriging predictions at the location and specific time lags prior to diagnosis for each individual. In addition, we examine the interaction between NDS and cancer stage at diagnosis. Figure 7 presents the stage-specific acceleration factor with 95% CI for different time lag windows (stages 1‐3, left to right). For instance, the estimated acceleration factors (95% CI) for averaged NDS 6 months prior to diagnosis for stages 1‐3 are 1.012 (0.376-2.720), 0.628 (0.436-0.904), and 0.976 (0.725-1.313), respectively. Notably, the negative effect of prediagnosis disinvestment on survival time is statistically significant only for stage 2 patients, with acceleration factor <1 for all time lags. In contrast, the acceleration factor is not significantly different from 1 for stage 1 or stage 3 patients. In Table S4 and Figure S2 in Multimedia Appendix 1, we include exploratory and sensitivity analysis (through multiple imputations implemented using R package mice) about the effect of missingness on survival modeling. To summarize, the proportions of missingness are comparable between low NDS and high NDS group (split by the median) for all the covariates included in the model ( “Exposure Time Lag Validation” section). Also, the stage-specific effect of NDS on CRC survival is very similar to the results based on complete cases reported in Figure 7.

## Discussion

### Principal Findings

This study addressed 3 questions related to the longitudinal assessment of built environment disinvestment; a potentially important exposure with growing evidence for a role in numerous health behaviors and outcomes [2,4]. First, historical GSV imagery was available to support repeated sampling across locations and dates, although uneven image availability and spatiotemporal clustering limited dense temporal sampling at some locations. Second, neighborhood disinvestment varied substantially across space but more modestly across time, with measurable correlation ranging approximately 2.8 kilometers spatially and 270 days temporally. Spring and summer have lower neighborhood disinvestment than fall. The traditional spatiotemporal regression-Kriging model using precise geographic locations achieved greater out-of-sample predictive accuracy than the physical boundary-based model, mean-trend-only prediction, and spatial-only Kriging alternatives. Third, greater prediagnosis neighborhood disinvestment was associated with shorter survival among individuals diagnosed with regional stage CRC but not among those diagnosed with localized or distant stage CRC; survival time ratios changed little across exposure windows. Together, these results support the feasibility and predictive value of modeling neighborhood disinvestment jointly across space and time, while indicating that its association with CRC survival differs by stage at diagnosis and is similar across selected prediagnosis exposure windows.

### Spatiotemporal Audit Sampling

While over 60% of audit locations had 2 or more eligible dates to audit, only approximately 2% had 5 eligible dates. Moreover, trellis plots of included audits indicate that location-dates may be spatiotemporally patterned; most timepoint-specific audits appear to derive from a specific subregion within the study region as opposed to a spatially random or uniform sample throughout the larger region. Such patterns could create difficulties in empirically separating spatial from temporal trends—especially small-scale trends—in the data. Moreover, we found a lower density of eligible dates toward the region’s periphery, which together with spatiotemporal patterns in data availability is consistent with prior findings [39].

### Spatiotemporal Variation and Model Validation of Neighborhood Disinvestment

We expected the model incorporating physical boundaries into estimates to outperform the conventional spatiotemporal Kriging model owing to face validity and empirical research demonstrating social stratification changes (eg, racial-ethnic segregation) when traversing major roadways, waterways, or railways [26,27]. However, our results provide evidence that the conventional spatiotemporal regression Kriging model had higher accuracy than the model incorporating boundaries. While some abrupt changes in disinvestment could follow major physical boundaries in the study area, results suggest that smoother changes in disinvestment with respect to distance and time offer a better explanation of the data. Within the traditional Kriging model, neighborhood disinvestment exhibited both small- and large-scale spatiotemporal variability, with the majority varying across space compared with time. Across larger temporal scales, disinvestment increased slightly over the study period and both spring and summer yielded coefficients indicative of lower disinvestment compared with fall. In terms of smaller-scale variation, disinvestment values exhibit small but measurable temporal variation (semivariance=0.04) and a substantial amount of small-scale spatial variation (semivariance=0.24), such that disinvestment autocorrelation was measurable temporally within 270 days and spatially within 2.8 kilometers. These spatiotemporal model parameters should prompt future audit designs that sample spatially proximate locations across seasons of the year as well as dates within 270 days in order to generate more accurate disinvestment exposure estimates of participants in public health studies.

These spatial variability and autocorrelation magnitudes are comparable with other studies in various major cities and urban areas throughout the United States [1,15]. While few studies have explicitly tested neighborhood disinvestment temporal variation and autocorrelation [40], our findings of small to moderate temporal variation is in line with a recent study that reported κ coefficients for disinvestment audit items comparing earliest available and most recent images of the same location in back-to-back assessments conducted by the same rater [41]. The 5 disinvestment indicators and κ coefficients overlapping that of this study—buildings needing repairs (0.22), boarded or abandoned buildings (0.24), burned buildings (0.44), garbage or litter (0.43), and graffiti (0.64)—suggested “fair” to “substantial” agreement across a median image date separation of 10.5 years. For the typical public health study’s spatial and temporal scale (eg, city or larger spatially and 1‐10 years of exposure assessment temporally) and meaningful spatiotemporal measurement units (eg, tens of meters spatially and accumulations across years temporally), results of this study add to growing evidence indicating that neighborhood disinvestment varies more across space than time. On average, built environment change across time is incremental such that street-level visual transformations may not be apparent for many years. These findings can be useful in designing future spatiotemporal audits, indicating that lower-density temporal sampling and higher-density spatial sampling will yield more accurate neighborhood disinvestment exposure estimates than the opposite.

### Exposure Time Lags and Associations Between Neighborhood Disinvestment and CRC Survival

Our findings that neighborhood disinvestment as associated with survival only among those diagnosed at regional stage (stage 2) are consistent with previous studies of neighborhood disinvestment and breast cancer survival [1]. Paralleling our findings of small temporal variation in neighborhood disinvestment, there was little variation in the disinvestment—cancer survival associations as a function of exposure lags. Within each stage at diagnosis, the 95% CIs for survival time ratios are all heavily overlapped (comparing vertically within each subplot in Figure 7). The 95% CI lower bounds, however, for the longer duration exposure time lags were slightly further away from null, suggestive of better fitting associations when averaging disinvestment values 6-24 months prior to diagnosis. These small exposure lag variations could be spurious but, if true, could be due to better estimation of individuals’ disinvestment exposures over the course of multiple seasons. Central Ohio experiences 4 seasons with large variation in temperature, precipitation, and vegetation, which in turn could influence outdoor activity, perceptions of streetscapes, and experiences of stress.

### Strengths and Limitations

A main limitation of this explicit spatiotemporal analysis is the lack of residential mobility patterns of CRC cases used in the external validation analysis. This potentially important temporal dimension prevented full assessment of the spatiotemporal models’ performance. As previously mentioned, emerging evidence suggests that between 20% and 40% of cancer cases change residence both pre- and postdiagnosis, depending on follow-up length, age, and several other factors [18,20,42]. We were also limited in our ability to adjust for other potential confounders, including CRC screening, socioeconomic factors, and social cohesion, that are not available in cancer registry data. Franklin County is likely not representative of US disinvestment or CRC, and similar to a previous study that found variation in spatial-only Kriging parameters across US cities of New York, Philadelphia, Detroit, and San Jose, results could be sensitive to spatiotemporal practices and policies that impact disinvestment (investment strategies, abandoned property handling, etc) [15,17,43]. Finally, variation in image availability may have affected measurement precision over space and time. However, a moderate to large sample of nearly 6000 audits across space and time, use of previously validated audit items and CANVAS audit platform, and testing of various neighborhood disinvestment spatiotemporal model parameters and frameworks, internal cross-validation, and exposure time lag testing within a single investigation are main strengths.

### Conclusions

Auditing various built environment characteristics using GSV across a uniform distribution of US urban locations and dates (2009‐2022) is possible especially if a maximum of 3 dates per location are desired. A traditional spatiotemporal regression Kriging model was more accurate at predicting NDSs than a model accounting for sharp changes due to physical roadway, waterway, and railway boundaries. Despite relatively small temporal compared with larger spatial variation in neighborhood disinvestment, disinvestment was appreciably lower in spring and summer than in autumn and increased modestly over the 14-year period. Cancer stage–dependent associations between disinvestment and survival were slightly more precise when averaging 6‐24 months prior to diagnosis. In summary, built environment disinvestment exhibits appreciable variation over time, indicating a need for assessments across dates in addition to locations in order to capture important longitudinal trends.

## Supplementary material

10.2196/86279 Multimedia Appendix 1 Supplemental materials about audit item details, fixed effect spatial cluster model, kriging residuals by date quantiles, and exploratory and sensitivity analysis for covariate missingness in accelerated failure time model.

## Abbreviations

AIC: Akaike information criterion
BIC: Bayesian information criterion
CANVAS: Computer Assisted Neighborhood Visual Assessment System
CRC: colorectal cancer
GSV: Google Street View
IRB: institutional review board
NDS: neighborhood disinvestment score
RMSPE: root mean square prediction error
TIGER: Topologically Integrated Geographic Encoding and Referencing
