# Supplementary material for: Built Environment Audits Across Space and Time for Estimating Time-Varying Exposures: Cohort Study
Source: JMIR Form Res. 2026 Aug 19;10:e86279. doi: 10.2196/86279 (PMC13489288; doi:10.2196/86279)
Supplement: Supplementary file 1 [file formative-v10-e86279-s001.doc]

**Multimedia Appendix 1**

**Table S1.** Item Details and Response Frequency of Neighborhood Characteristics Audited in Central Ohio, 2022‒2023.

| **Audit question** | **Summary** | **Categories** | **N (%)** |
| --- | --- | --- | --- |
| Is the streetscape appropriate for auditing? | Appropriate streetscape | Yes vs no | 5772 (100.0) |
| **Date Related** |  |  |  |
| What is the earliest Year, 2009+? | Earliest year | No back-in-time imagery | 0 (0.0) |
|  |  | 2009 | 1031 (54.0) |
|  |  | 2010 | 0 (0.0) |
|  |  | 2011 | 304 (15.9) |
|  |  | 2012 | 180 (9.4) |
|  |  | 2013 | 0 (0.0) |
|  |  | 2014 | 45 (2.4) |
|  |  | 2015 | 116 (6.1) |
|  |  | 2016 | 20 (1.1) |
|  |  | 2017 | 1 (0.1) |
|  |  | 2018 | 1 (0.1) |
|  |  | 2019 | 210 (11.0) |
|  |  | 2020 | 0 (0.0) |
|  |  | 2021 | 1 (0.1) |
|  |  | 2022 | 0 (0.0) |
| Which year approximates Q1? | Approximate 1^st^ quarter year | No eligible date | 0 (0.0) |
|  |  | 2009 | 9 (1.3) |
|  |  | 2010 | 0 (0.0) |
|  |  | 2011 | 203 (29.9) |
|  |  | 2012 | 151 (22.3) |
|  |  | 2013 | 0 (0.0) |
|  |  | 2014 | 186 (27.4) |
|  |  | 2015 | 95 (14.0) |
|  |  | 2016 | 16 (2.4) |
|  |  | 2017 | 4 (0.6) |
|  |  | 2018 | 1 (0.2) |
|  |  | 2019 | 13 (1.9) |
|  |  | 2020 | 0 (0.0) |
|  |  | 2021 | 0 (0.0) |
|  |  | 2022 | 0 (0.0) |
| Which year approximates the midpoint? | Midpoint year | No eligible date | 0 (0.0) |
|  |  | 2009 | 0 (0.0) |
|  |  | 2010 | 0 (0.0) |
|  |  | 2011 | 4 (0.6) |
|  |  | 2012 | 10 (1.5) |
|  |  | 2013 | 0 (0.0) |
|  |  | 2014 | 139 (21.1) |
|  |  | 2015 | 307 (46.6) |
|  |  | 2016 | 81 (12.3) |
|  |  | 2017 | 63 (9.6) |
|  |  | 2018 | 10 (1.5) |
|  |  | 2019 | 44 (6.7) |
|  |  | 2020 | 0 (0.0) |
|  |  | 2021 | 1 (0.2) |
|  |  | 2022 | 0 (0.0) |
| Which year approximates Q3? | Approximate 3^rd^ quarter year | No eligible date | 0 (0.0) |
|  |  | 2009 | 0 (0.0) |
|  |  | 2010 | 0 (0.0) |
|  |  | 2011 | 0 (0.0) |
|  |  | 2012 | 1 (0.2) |
|  |  | 2013 | 0 (0.0) |
|  |  | 2014 | 26 (4.3) |
|  |  | 2015 | 145 (24.2) |
|  |  | 2016 | 24 (4.0) |
|  |  | 2017 | 30 (5.0) |
|  |  | 2018 | 33 (5.5) |
|  |  | 2019 | 271 (45.2) |
|  |  | 2020 | 48 (8.0) |
|  |  | 2021 | 21 (3.5) |
|  |  | 2022 | 1 (0.2) |
| What is the most recent Year, 2009+? | Most recent year | No eligible date | 0 (0.0) |
|  |  | 2009 | 0 (0.0) |
|  |  | 2010 | 0 (0.0) |
|  |  | 2011 | 2 (0.1) |
|  |  | 2012 | 6 (0.3) |
|  |  | 2013 | 0 (0.0) |
|  |  | 2014 | 12 (0.6) |
|  |  | 2015 | 96 (5.0) |
|  |  | 2016 | 3 (0.2) |
|  |  | 2017 | 2 (0.1) |
|  |  | 2018 | 2 (0.1) |
|  |  | 2019 | 634 (32.9) |
|  |  | 2020 | 90 (4.7) |
|  |  | 2021 | 413 (21.5) |
|  |  | 2022 | 665 (34.6) |
| Which month is associated with the year? | Associated month | Winter: Jan-Mar | 41 (0.7) |
|  |  | Spring: Apr-Jun | 1468 (25.4) |
|  |  | Summer: Jul-Sep | 3028 (52.5) |
|  |  | Fall: Oct-Dec | 1234 (21.4) |
| **Physical disorder/Disinvestment** |  |  |  |
| Is there garbage, litter, or broken glass in the street or on the sidewalks (≥5 items)? | Garbage, litter, or broken glass | Yes vs no | 689 (11.9) |
| Is there graffiti, or evidence of graffiti that has been painted over, on buildings, signs, or walls? | Graffiti | Yes vs no | 175 (3.0) |
| Do you see boarded/abandoned or burned-out buildings? | Abandoned or burned-out buildings | Yes vs no | 242 (4.2) |
| What is the poorest building condition? | Poorest building condition | Very well kept/good condition | 351 (6.4) |
|  |  | Moderately well kept condition | 3879 (70.5) |
|  |  | Fair condition | 959 (17.4) |
|  |  | Poor/badly deteriorated condition | 315 (5.7) |
| What is the poorest yard condition? | Poorest yard condition | Very well kept/good condition | 201 (3.6) |
|  |  | Moderately well kept condition | 1887 (33.7) |
|  |  | Fair condition | 1948 (34.8) |
|  |  | Poor/badly deteriorated condition | 1568 (28.0) |
| Are there dumpsters visible? | Dumpsters | Any vs none | 554 (9.6) |
| **Engagement** |  |  |  |
| Do you see any exterior or yard decorations? | Yard decoration | Yes vs no | 3451 (60.0) |
| Do you see any team-sports equipment in public spaces? | Team-sports equipment | Yes vs no | 125 (2.2) |
| Do you see any potted plants? | Potted plants | Yes vs no | 2734 (47.5) |
| Do you see any outdoor seating? | Outdoor seating | Yes vs no | 2763 (47.9) |
| What is the poorest road verge condition? | Poorest road verge condition | Very well kept/good condition | 93 (2.5) |
|  |  | Moderately well kept condition | 1336 (35.9) |
|  |  | Fair condition | 1627 (43.7) |
|  |  | Poor/badly maintained | 664 (17.9) |
| **Sidewalk walkability** |  |  |  |
| What type of sidewalk or path (paved or unpaved) is there? | Sidewalk type | Sidewalk vs none | 3986 (69.1) |
| What is the poorest sidewalk or pedestrian path condition? | Poorest sidewalk or pedestrian path condition | Good | 2973 (75.0) |
|  |  | Fair | 804 (20.3) |
|  |  | Poor | 188 (4.7) |

## **Fixed effect spatial cluster model**

*Model 2-F*. Consider a fixed effect model including spatial cluster, year and seasonality:

|  | (1) |
| --- | --- |

and are the same as those defined in Section 2.6.1 of the main paper. represents which spatial cluster location belongs to. Cluster is modeled using a categorical variable with 530 levels, cluster 1 as the reference level. Therefore, the coefficient vector has length 529, representing the relative cluster effect compared to cluster 1.

### **In sample mean trend model fit**

Table S2. AIC and BIC for Model 2-F, full and reduced (without season).

| Models | AIC | BIC |
| --- | --- | --- |
| Model 2-F | 11619.145 | 15180.709 |
| Model 2r-F | 11653.931 | 15195.524 |

Table S3. Coefficients, test statistics and P values for Model 2-F fixed effects.

| Fixed effect | coefficient | t value | *P* value |
| --- | --- | --- | --- |
| season =1 | 0.009 | 0.079 | .94 |
| season =2 | -0.165 | -6.011 | <.001 |
| season =3 | -0.088 | -3.774 | <.001 |
| time | 0.006 | 2.974 | .003 |

Note: For the cluster effect estimates, there are 61.6% (326/529) clusters that have significant effects (*P* value < .05) on NDS.

### **Out-of-sample sample prediction accuracy**

For Model 2-F, the out-of-sample RMSPE and standard error (in parenthesis) for mean trend only and kriging prediction are 0.679 (SE 0.002) and 0.671 (SE 0.002), respectively.

Figure S1: Kriging residuals partitioned by date quantiles.

As an example, Panel 1 (earliest) includes samples whose audit dates are the earliest at each specific location. Residuals may exhibit spatial clustering effects, where adjacent points tend to have similar residual values. However, model 2-R model error (Table 3 of main manuscript) which accounted for nearby NDS scores via random effects for discrete neighborhoods that do not cross physical barriers was higher than Model 1 (traditional Kriging, residuals shown above), suggesting that residual spatial clustering is not captured by these neighborhoods nor spatio-temporal Kriging of residuals.

### **Exploratory and sensitivity analysis for covariate missingness in survival modeling**

Table S4 summarizes the empirical distribution of the categorical covariates (percentage of each category) we included in the survival model, as well as the proportion of missingness, split by low versus high NDS at diagnosis (low NDS means NDS is smaller than the median of all patients’ NDS at diagnosis, and high NDS means greater than the median). The percentages of missingness for all of the covariates are comparable for low versus high NDS group, suggesting that there is no evidence of severe imbalanced missingness. There is no missingness in sex and age.

To investigate the effect of missingness on survival modeling results, we conducted sensitivity analysis using Multiple Imputation by Chained Equations (MICE), implemented using R package mice. We generated 10 imputed, complete datasets, fitting the AFT model on each dataset, and pooled the estimates based on Rubin’s rules.[1] The stage-specific effect of NDS on CRC survival for each time lag and their 95% Cis using the pooled estimates are shown in Figure S2. Compared to Figure 7 in the main manuscript which only uses complete cases, stage 1 (localized) CIs become tighter, whereas stage 2 and 3 CIs are very similar to those in Figure 7. The conclusions stay the same: higher pre-diagnosis NDS was significantly associated with shorter survival only among those with regional and not among those with localized or distant stage at diagnosis. This indicates the exposure validation results are robust to a moderate proportion of missingness (13.2%) in our data.

Figure S2: Stage-specific effect of NDS on CRC survival using MICE generated datasets. Results pooled by Rubin’s rules.

Table S4. Distribution of categorical covariates and their missingness by low and high NDS

| **Characteristic** | **NDS Low**  N = 1,364^1^ | **NDS High**  N = 1,363^1^ |
| --- | --- | --- |
| Primary insurance |  |  |
| Uninsured | 48 (3.5%) | 67 (4.9%) |
| Medicaid | 74 (5.4%) | 201 (15%) |
| Other | 27 (2.0%) | 56 (4.1%) |
| Medicare | 613 (45%) | 554 (41%) |
| Private | 519 (38%) | 398 (29%) |
| Missing | 83 (6.1%) | 87 (6.4%) |
| Race/ethnicity |  |  |
| White | 1,079 (79%) | 890 (65%) |
| Black | 148 (11%) | 367 (27%) |
| Non-White/Black | 53 (3.9%) | 32 (2.3%) |
| Latino | 16 (1.2%) | 17 (1.2%) |
| Missing | 68 (5.0%) | 57 (4.2%) |
| Marital/partner status |  |  |
| S/D/W/Never Married | 536 (39%) | 733 (54%) |
| Married/Partnered | 708 (52%) | 502 (37%) |
| Missing | 120 (8.8%) | 128 (9.4%) |
| Stage at diagnosis |  |  |
| Localized | 432 (32%) | 371 (27%) |
| Regional | 555 (41%) | 558 (41%) |
| Distant | 267 (20%) | 320 (23%) |
| Missing | 110 (8.1%) | 114 (8.4%) |
| Sex |  |  |
| Male | 668 (49%) | 728 (53%) |
| Female | 696 (51%) | 635 (47%) |
| Age at diagnosis | 63 (15) | 62 (14) |
| ^1^n (%); Mean (SD) | | |

1.	Rubin DB. Multiple imputation for nonresponse in surveys. New York ; Chichester: Wiley; 1987. xxix,258p : ill. p. ISBN: 047108705X.
